# Outlines of a multiple trace theory of temporal preparation

**DOI:** 10.3389/fpsyg.2014.01058

**Published:** 2014-09-19

**Authors:** Sander A. Los, Wouter Kruijne, Martijn Meeter

**Affiliations:** Department of Cognitive Psychology, VU University AmsterdamAmsterdam, Netherlands

**Keywords:** temporal preparation, trace conditioning, foreperiod effects, multiple trace theory, hazard function

## Abstract

We outline a new multiple trace theory of temporal preparation (MTP), which accounts for behavior in reaction time (RT) tasks in which the participant is presented with a warning stimulus (S1) followed by a target stimulus (S2) that requires a speeded response. The theory assumes that during the foreperiod (FP; the S1–S2 interval) inhibition is applied to prevent premature response, while a wave of activation occurs upon the presentation of S2. On each trial, these actions are stored in a separate memory trace, which, jointly with earlier formed memory traces, starts contributing to preparation on subsequent trials. We show that MTP accounts for classic effects in temporal preparation, including mean RT–FP functions observed under a variety of FP distributions and asymmetric sequential effects. We discuss the advantages of MTP over other accounts of these effects (trace-conditioning and hazard-based explanations) and suggest a critical experiment to empirically distinguish among them.

## INTRODUCTION

Timing the occurrence of future events is a fundamental ability that we routinely apply in a wide variety of activities, such as driving a car, cooking a meal, jumping up to catch a ball, or drawing up our daily agenda. In all these activities, we make use of temporal contingencies that we have learned during our lives and that we continuously update on the basis of new experiences. For instance, we are reasonably accurate in predicting when the traffic light will turn green given the time that has elapsed since we had to stop our car in front of it. If we have to wait longer than we normally do, it is only a matter of seconds before we start wondering whether the traffic light might be out of order. However, if the unexpected delay is due to a changed phasing of the traffic light, our subsequent exposures to it will gradually bring our expectancies in sync again with the new time regime.

Accurate timing is important because it may facilitate several stages of information processing (Nobre and Coull, 2010) across a wide variety of tasks, such as reaction time (RT) tasks (e.g., Niemi and Näätänen, 1981), perceptual identification (e.g., Rolke, 2008; Rohenkohl et al., 2012; Vangkilde et al., 2013), attentional selection (e.g., Yashar and Lamy, 2013; Seibold and Rolke, 2014), temporal discrimination (e.g., Grondin and Rammsayer, 2003; Los and Horoufchin, 2011), and time reproduction (e.g., Jazayeri and Shadlen, 2010; Taatgen and Van Rijn, 2011). In the present article, we focus on timing processes in the RT task, but our theoretical analysis may be revealing about the nature of timing processes in other task domains as well, since it ranges over general cognitive principles of learning and memory.

In RT tasks, it has been shown that participants respond faster to a target stimulus (S2) when it is preceded by a neutral warning stimulus (S1; e.g., a brief sound) than when it is presented alone (e.g., Niemi and Näätänen, 1981; Hackley, 2009). This old experimental finding (e.g., Woodrow, 1914) has led to the postulation of a process of temporal preparation^1^that develops during the foreperiod (FP), the interval between the offset of S1 and the onset of S2. Research in which FP has been systematically varied indicates that temporal preparation may develop very quickly and reach an optimum in less than half a second (e.g., Müller-Gethmann et al., 2003). However, the time course of temporal preparation is by no means fixed, as it is tuned by the FPs that occurred on earlier trials during the experimental session. In particular, the development of temporal preparation during FP on any given trial is modified both by the FPs occurring on the immediately preceding trials (a short-term effect; e.g., Los and Van den Heuvel, 2001; Vallesi and Shallice, 2007) and by the wider context of FPs in the experimental session (a long-term effect; e.g., Trillenberg et al., 2000; Janssen and Shadlen, 2005).

These effects suggest that temporal preparation is driven by two mechanisms, operating at different time scales. Thus, Vallesi and Shallice (2007) proposed a dual-process model, in which the short-term effect is attributed to an automatic process and the long-term effect to a controlled process. However, in this article we will show that these effects, while having distinctive signatures, may still proceed from a common implicit learning mechanism. The central idea of our new multiple trace theory of temporal preparation (MTP), is that both effects result from the joint activation of memory traces created on a trial-by-trial basis during an experimental session. This multiple-trace conception has recently been successfully applied in the literature on time perception (Taatgen and Van Rijn, 2011), and we will show that it is promising in explaining the phenomena of temporal preparation, too.

In what follows, we first describe a set of interrelated experimental findings that we believe to be crucial to understanding the processes underlying temporal preparation. Next, we discuss two fundamental explanatory constructs: the hazard function (e.g., Luce, 1986; Nobre et al., 2007) and trace conditioning (e.g., Los and Van den Heuvel, 2001; Steinborn et al., 2009). It transpires that the scope and limitations of these constructs are complementary. The hazard function is well equipped to deal with long-term effects in temporal preparation, but fails to address the short-term effects, while the situation is reversed for trace conditioning. Next, we discuss Vallesi and Shallice’s (2007) dual-process model that maintains a role for the hazard function and extends it with a short-term component. Although this model accounts for many of the reported findings, we will argue that it relies on disputable assumptions. Then, we outline MTP, which we develop as an extension of the trace-conditioning model. Finally, we evaluate MTP and specify a possible empirical test.

## A FAMILY OF PHENOMENA IN TEMPORAL PREPARATION

The phenomena of temporal preparation that we describe in this section are all taken from RT tasks, in which participants are instructed to prepare on the basis of S1 (a mere time marker, such as a brief tone) and to respond as quickly as possible to S2. Across these studies, strong, and consistent influences of FP duration and FP context have been demonstrated, regardless of other details of experimental design, such as the modality of S1 or S2 (e.g., auditory or visual; e.g., Niemi and Näätänen, 1981; Müller-Gethmann et al., 2003; Steinborn et al., 2009) or the task with respect to S2 (e.g., simple or choice; Niemi and Näätänen, 1981; Los and Van den Heuvel, 2001; Müller-Gethmann et al., 2003).

**FIGURE 1:**
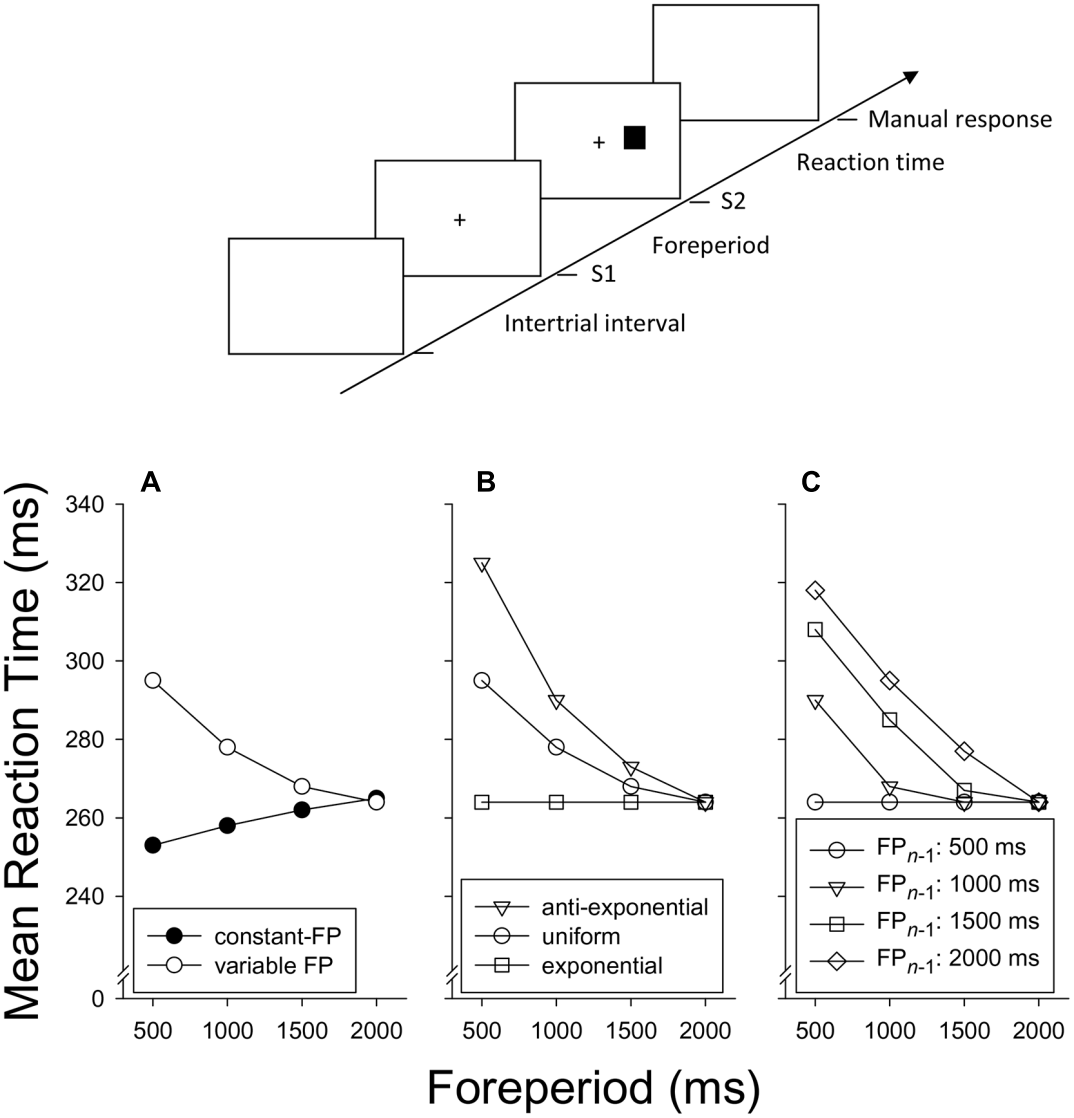
**Top.** Course of events on a single trial in a typical foreperiod (FP) paradigm. Here, the neutral warning stimulus (S1) and the target stimulus (S2) are both visual and the location of S2 (left or right of fixation) requires a spatially compatible manual choice response. **Bottom**. A family of idealized data patterns. **(A)** Mean reaction time as a function of FP in the constant-FP and the variable-FP paradigm (uniform FP-distribution). **(B)** Mean reaction time as a function of FP in the variable-FP paradigm for different distributions of FP. **(C)** Mean reaction time in the variable-FP paradigm (uniform distribution), as a function of FP on trial *n* and FP on trial *n* - 1.

As a standard experimental design, consider that FP is varied at four possible levels, of 500, 1000, 1500, and 2000 ms (see **Figure 1**, top panel for details). In the constant-FP paradigm, these FPs are presented in separate blocks of trials, such that FP remains constant within a block and varies between blocks. In the variable-FP paradigm, FP is sampled on a trial-by-trial basis from a certain distribution of FPs. For instance, in the case of a uniform FP-distribution, each particular FP of our standard design has a 1/4 probability of being sampled on each trial. Furthermore, we define a possible moment of S2 presentation relative to the offset of S1 as a *critical moment*. The moment that is used for the presentation of S2, on any particular trial, we define as the *imperative moment* of that trial. That is, in the constant-FP paradigm, there is only one critical moment, which is the imperative moment on each trial. In the variable-FP paradigm, there are several critical moments (four in our standard design), of which one will be the imperative moment on any particular trial (see **Table 1** for an overview of definitions).

**Table 1:** Overview of definitions.

Concept	Definition
S1	Warning stimulus that serves as a mere time marker for the occurrence of S2
S2	Target stimulus that requires a speeded response
Foreperiod (FP)	Inter stimulus interval between S1 and S2
Critical moment	A possible moment of S2 presentation relative to the offset of S1
Imperative moment	The moment of S2 presentation, on any given trial, relative to the offset of S1
MTP	Multiple trace theory of temporal preparation
Trace weight	The strength of a memory trace in its contribution to temporal preparation
AI-ratio	Hypothetical ratio between activation and inhibition values across memory traces

**Figure 1A** shows mean RT as a function of FP as typically observed in the constant-FP paradigm and the variable-FP paradigm with a uniform distribution (e.g., Woodrow, 1914; Los and Van den Heuvel, 2001; Los et al., 2001)^2^. Clearly, with an increase of FP, mean RT increases in the constant-FP paradigm, but decreases in the variable-FP paradigm, typically according to an exponential decay function. These RT–FP functions converge toward the longer FPs, such that no difference remains at the longest FP. The striking difference between these functions may easily obscure that they are in fact members of the same family. To see this, consider the effect of the following FP distributions (or their discrete counterparts) in the variable-FP paradigm: uniform, exponential (i.e., when FPs occur more frequently as they are shorter), and anti-exponential (i.e., when FPs occur more frequently as they are longer). Typical data are shown in **Figure 1B** (e.g., Zahn and Rosenthal, 1966; Baumeister and Joubert, 1969; Näätänen, 1970, 1971; Trillenberg et al., 2000). Clearly, as the relative frequency of short FPs increases, the FP–RT function gradually lies down and becomes approximately flat in the case of an exponential FP-distribution (e.g., Näätänen, 1971; Trillenberg et al., 2000; Vangkilde et al., 2013). By extrapolation, it can be inferred that the RT–FP function will end up upward sloping when the probability of each FP is set to 1 in different blocks, as is the case in the constant-FP paradigm.

The last member of the family of FP phenomena is the asymmetric sequential effect of FP. This effect can be observed in the variable-FP paradigm when RT on any trial *n* is analyzed, not only as a function of FP on that trial (FP*_n_*), but also of FP on the immediately preceding trial (FP*_n_*_-1_). As **Figure 1C** shows, the typical pattern is that RT is longer to the extent that FP*_n_*_-1_ is longer than FP*_n_*. As a result, for the shortest FP*_n_*, RT strongly increases as FP*_n_*_-1_ increases. This dependence on FP*_n_*_-1_ becomes less as FP*_n_* increases, and eventually disappears for the longest FP*_n_* (e.g., Woodrow, 1914; Karlin, 1959; Drazin, 1961; Zahn et al., 1963; Los and Van den Heuvel, 2001; Van der Lubbe et al., 2004; Vallesi and Shallice, 2007; Steinborn and Langner, 2012). Of note is the relationship of the asymmetrical sequential effect with the FP-distribution effect (**Figure 1B**). If the relative frequency of short FPs increases, short FPs are increasingly more often preceded by short FPs and increasingly less often by long FPs. As a result, favorable FP transitions (in terms of RT) are increasingly more frequent for the short FPs, which in turn should contribute to a flatter RT–FP function, averaged across FP*_n_*_-1_.

Finally, it is worth noting that these typical effects break down for brief FP durations and for brief ranges of FPs. If FP is varied at brief intervals up to about 300 ms, RT decreases exponentially as a function of FP, not only in the variable-FP paradigm (e.g., Los and Van der Burg, 2013) but also in the constant-FP paradigm (e.g., Los and Schut, 2008). This finding is consistent with the idea that temporal preparation is not an instantaneous process, but takes time to develop. Thus, in the constant-FP paradigm, the RT–FP function initially decreases sharply, reaches its nadir by about 300 ms, after which it increases slowly (**Figure 1A**) until it levels off toward 20 s (e.g., Woodrow, 1914; Klemmer, 1956; Müller-Gethmann et al., 2003; Lawrence and Klein, 2013). Furthermore, if the range of FP durations is small relative to the mean duration of FP, the RT–FP function becomes less pronounced and sequential effects have been shown to deviate from the typical pattern shown in **Figure 1C** (Karlin, 1959; Steinborn et al., 2008). These deviations probably reflect the inaccuracy of our mental timekeeping system, which has been shown to scale with the duration of FP following a Weber fraction (e.g., Klemmer, 1957; Gibbon, 1977; Roberts, 1981; Gallistel and Gibbon, 2000).

In the present contribution, the focus is on the family of FP effects shown in **Figure 1**, which we consider to be most revealing about the learning principles underlying temporal preparation. The just noted deviations seem less important in this respect, although they may be informative about the nature of the timing mechanism involved.

## TWO EXPLANATORY CONSTRUCTS

### THE HAZARD FUNCTION

The hazard function describes the development of the conditional probability that S2 will occur at the next critical moment, given that it has not yet occurred. Thus, in our standard design with a uniform FP distribution, the hazard is 1/4 for the first critical moment at the very beginning of FP. If the first moment is passed without occurrence of S2, the hazard increases to 1/3 for the second critical moment, because three equiprobable critical moments remain. If this moment is also passed without S2 occurrence, it increases further to 1/2 for the third critical moment. Finally, if S2 has still not occurred by that time, the participant can be certain that S2 will occur at the last critical moment, and the hazard thus becomes 1. In the anti-exponential distribution, this increasing hazard over subsequent critical moments is even more dramatic, whereas in the exponential (“non-aging”) distribution it has a constant value across critical moments (e.g., 1/2)^3^. Finally, the hazard is also constant in the constant-FP paradigm, where its value is fixed at 1.

In itself, the hazard function is just a statistical fact determined by the FP distribution, but its merit as an explanatory construct of FP phenomena has been recognized since the earliest reports on variable FP phenomena (Woodrow, 1914; for recent contributions, see e.g., Janssen and Shadlen, 2005; Nobre et al., 2007; Coull, 2009; Cui et al., 2009; Vangkilde et al., 2012, 2013). The general idea is that hazard drives temporal preparation (or the expectancy of S2 presentation) and thus comes indirectly to expression in the RT–FP function. In a detailed version of this idea, temporal preparation is considered to be an effortful process that can be maintained at an optimal level for only a very brief period of time (e.g., Näätänen, 1971; Gottsdanker, 1975; Jennings and Van der Molen, 2005). As a result, participants optimize their state of preparation in keeping with the hazard function, so as to minimize the time spent in idle preparation (Vallesi and Shallice, 2007). Although in most writings the causal chain leading from hazard to the RT–FP function remains implicit, some version has to be endorsed, since otherwise hazard would be an essentially meaningless statistical fact in the literature of temporal preparation.

A major reason why the hazard function has found general acceptance as a valid explanatory construct is that it well explains the shape of the RT–FP functions in the variable-FP paradigm (e.g., Niemi and Näätänen, 1981; Luce, 1986; Coull, 2009). As can be easily verified, mean RT in **Figure 1B** is shorter as the hazard at the imperative moment is higher, and in the exponential FP-distribution, the constant hazard co-occurs with an approximately flat RT–FP function (e.g., Näätänen, 1971; Trillenberg et al., 2000; Vangkilde et al., 2013). This strongly suggests that temporal preparation is driven by hazard: The higher the hazard, the higher the preparatory state.

Although the hazard function excellently accounts for the mean RT–FP functions in the variable-FP paradigm, it fails remarkably in accounting for the other phenomena of the family. First, the hazard function completely fails to explain the asymmetric sequential effect of FP (**Figure 1C**). The hazard function is fixed for any specific FP distribution, so it should drive temporal preparation in the same way on each and every trial of a block, that is, independent of the FP that occurred on the preceding trial. Second, the hazard is constant for each critical moment in both the constant-FP paradigm (a value of 1) and the variable-FP paradigm with an exponential FP-distribution (a value of, e.g., 1/2), yet the slopes of the corresponding RT–FP functions are not the same (cf. **Figures 1A,B**). In the constant-FP paradigm, the upward-sloping RT–FP function has invariably been attributed to time uncertainty (caused by an imperfect time keeping device), which increases in proportion with the duration of FP (e.g., Klemmer, 1956, 1957; Müller-Gethmann et al., 2003). This inference relies on the fact that the hazard for each critical moment is constant, such that any source of uncertainty other than time uncertainty is controlled for. However, the hazard for each critical moment is also constant for non-aging FPs (i.e., an exponential FP-distribution), so one would expect the RT–FP function in this condition to be also upward sloping instead of flat. That is, the approximately flat RT–FP function for non-aging FPs is problematic for the view of hazard-driven preparation rather than support for it, as often claimed.

Apart from these empirical problems, there is also a theoretical problem that deserves consideration. As the hazard function by itself is devoid of psychological content, it remains to be specified how participants acquire knowledge about the changing hazard during FP. The literature is remarkably silent on this important issue. Since RT–FP functions similar to those observed in adults have been observed in young children (Elliott, 1970; Vallesi and Shallice, 2007) as well as in non-human mammals, such as monkeys and rodents (Janssen and Shadlen, 2005; Narayanan and Laubach, 2006; Tsunoda and Kakei, 2008), it seems clear that the critical parameters for constructing the hazard function must be acquired through experience rather than through instruction. This in turn raises questions about how experience is transformed into a hazard computation and moreover, on which sample of trials this inference is based. Ironically, if the sample is small or if recent trials receive a greater weight than earlier trials in a block, proponents of the hazard function will have to face the sequential effect of FP, which they have usually chosen to ignore.

### TRACE CONDITIONING

To avoid the problems with the hazard function, Los and Van den Heuvel (2001; see also, e.g., Los and Heslenfeld, 2005; Steinborn et al., 2009; Los, 2013) reasoned that it is crucial to come up first with an explanation of the asymmetric sequential effect of FP. This explanation would indirectly account for the other phenomena of the family by simple averaging across sequential effects. Obviously, the downward sloping RT–FP function of **Figure 1A** follows directly from averaging across FP*_n_*_-1_ (**Figure 1C**). By extension, the FP-distribution effect (**Figure 1B**) would reflect that intertrial repetitions of short FPs occur more frequently as the skew of the FP distribution becomes more positive, thus explaining the corresponding flattening of the average RT–FP function.

To account for the asymmetric sequential effect, Los and Van den Heuvel (2001) proposed a trace-conditioning model, based on learning principles described in the literature on animal timing (e.g., Bradshaw and Szabadi, 1997). This model assumes that participants act on the basis of a mental representation of the critical moments. In this representation, each critical moment is associated with a variable conditioned strength. The higher the conditioned strength of a critical moment, the faster the participant responds if S2 is presented at that critical moment. It is furthermore assumed that the response system is kept under inhibition during FP to prevent premature response, whereas a wave of activation is released when S2 is presented and responded to. These inhibitory and activating processes adjust the conditioned strength of each critical moment in accordance with three learning rules. First, as a critical moment is passed by during FP, its conditioned strength is reduced (extinction). For each critical moment, extinction gradually increases as that moment is approached, reaches its maximum as it is passed by and gradually reduces afterward. Second, when S2 is presented and responded to, the conditioned strength of critical moments is increased (reinforcement). Reinforcement is maximal at the imperative moment and tapers off toward earlier and later critical moments as they are more remote from the imperative moment. Third, the conditioned strength of critical moments beyond the imperative moment are left relatively unchanged, because they are neither passed by during FP, nor used for the presentation of S2.

**FIGURE 2:**
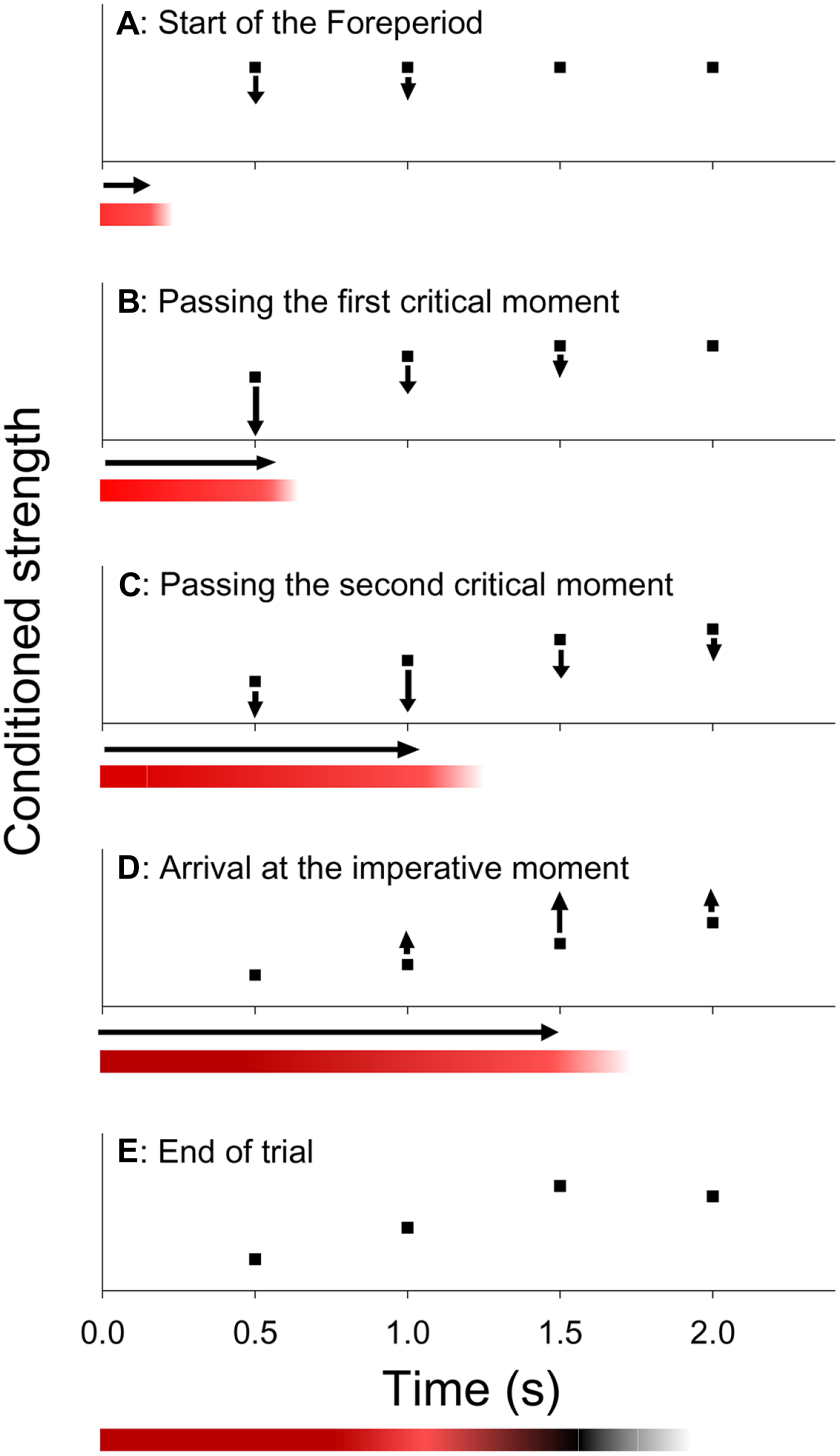
**Within trial dynamics according to the trace-conditioning model of temporal preparation (graphs) and the formation of a memory trace according to the multiple trace theory of temporal preparation (colored bars).** Graphs **(A)** Hypothetical state of conditioning at the start of a trial, showing an equal conditioned strength for each critical moment. **(B,C)** During the FP inhibition is applied, resulting in a reduction of the conditioned strength of critical moments as they are passed by during FP. **(D)** At the imperative moment, a wave of activation is released when S2 is presented and responded to, which results in an increase of the conditioned strength, especially at the imperative moment. **(E)** Final state of conditioning, which carries over to the next trial. Colored bars. The bars show the creation of a memory trace that represents the cumulative levels of inhibition (red shades) and activation (black shades) for each time point during FP.

**Figure 2** (graphs) illustrates the dynamics of this model. Suppose that at the start of a given trial, each critical moment has an equal conditioned strength (**Figure 2A**) and that, unbeknownst to the participant, the third critical moment is about to become the imperative moment of that trial. Now, as the first two critical moments are passed during FP, the conditioned strength associated with those moments is subject to extinction (**Figures 2B,C**). Subsequently, when S2 is presented and responded to, the conditioned strength of the imperative moment is increased (**Figure 2D**). Finally, the conditioned strength of the last critical moment is left relatively unchanged, because it is neither passed by during FP, nor used for the presentation of the S2. The state of conditioning that is reached after the response of the participant (**Figure 2E**) is preserved and carries over to the next trial. Thus, on the next trial, response is expected to be relatively slow when S2 is presented at the (just extinguished) first or second critical moment, and relatively fast when it occurs at the (just reinforced) third critical moment or at the (unchanged) last critical moment, consistent with the asymmetric sequential effect. Also note that the last critical moment is virtually immune to whatever FP occurred on the preceding trial, because its conditioned strength is never subject to appreciable extinction, such that it will be driven to ceiling after a few reinforcing trials (i.e., with S2 appearing at the last critical moment).

The core assumptions of this trace-conditioning model are consistent with (Machado’s (1997) formal model in the animal timing literature. Los et al. (2001; see also Los, 2013) adjusted this model and obtained a good fit when they applied it simultaneously to the pattern of asymmetric sequential effects in the variable-FP paradigm (**Figure 1C**) and the mean RT–FP functions in both paradigms (**Figure 1A**). Furthermore, the model is supported by a large body of evidence indicating that several areas of the central nervous system are kept under inhibition during FP (e.g., Hasbroucq et al., 1999; Prut and Fetz, 1999; Narayanan and Laubach, 2006; Van Elswijk et al., 2007). It has been argued that this inhibition serves to prevent a response from being emitted prematurely (e.g., Narayanan et al., 2006; Davranche et al., 2007; Duque and Ivry, 2009) and Los (2013) presented behavioral evidence for a causal link between this function and the extinction mechanism of the trace-conditioning model. Finally, several other studies have provided support for the model by showing that factors that are known to modify the conditioned response in animal timing also modify the sequential effect of FP, suggesting common underlying mechanisms (Steinborn et al., 2008, 2009, 2010; Langner et al., 2010).

Unfortunately, the model fares less well when considering the effect of FP distribution (**Figure 1B**). As noted earlier, the initial idea was that the FP-distribution effect is a simple consequence of the sequential effect, because shifts in the FP distribution co-occur with shifts in the relative frequency of specific intertrial transitions. However, closer examination reveals that the contribution of the sequential effect to the mean RT–FP function is insufficiently powerful to account for the full FP-distribution effect. As **Figure 1C** shows, the RT–FP function in the uniform FP-distribution is approximately flat after a short FP*_n_*_-1_ and more and more downward sloping as FP*_n_*_-1_ increases. In the exponential FP-distribution with a constant hazard of 1/2, half of the trials is preceded by the shortest FP*_n_*_-1_; the other half is preceded by a longer FP*_n_*_-1_. Therefore, the RT–FP function resulting after averaging across the effect of FP*_n_*_-1_ should still be downward sloping instead of flat, as typically observed (**Figure 1B**). Using a formal procedure, Los and Agter (2005) estimated the contribution of sequential effects to the FP-distribution effect, and established that it was below 20% in all conditions they examined. The bottom line is that the FP-distribution effect cannot be conceived as a simple propagation of the asymmetric sequential effect of FP (see also Drazin, 1961; Zahn and Rosenthal, 1966; Alegria and Delhaye-Rembaux, 1975; Possamaï et al., 1975).

More recent studies have further challenged the trace-conditioning model by showing that the coupling between the sequential effect of FP and the shape of the RT–FP function is loose at best. For instance, Vallesi et al. (2013) used the variable-FP paradigm with a uniform FP-distribution, and varied the intertrial interval. They observed that an increase of the intertrial interval attenuated the sequential effect and its asymmetry while leaving the average RT–FP function relatively unchanged. Conversely, Vallesi and Shallice (2007) observed that, across different age groups ranging from 4 years old up to adulthood, the average RT–FP function, under a uniform condition, became steeper whereas the sequential effect remained fairly stable (though not its asymmetry). These and similar dissociations between sequential effects and the average RT–FP function (e.g., Vallesi et al., 2007, 2009; Triviño et al., 2010; Steinborn and Langner, 2011; Capizzi et al., 2012) have clearly revealed the limitations of the trace-conditioning model.

## INTERIM SUMMARY: A SPLIT IN THE FAMILY?

The evidence just reviewed suggests that the family ties among FP phenomena may not be as strong as earlier assumed. Indeed, the rather loose coupling between the asymmetrical sequential effect of FP on the one hand and the mean RT–FP function and the FP-distribution effect on the other hand suggests a split in the family of FP phenomena along the lines of longevity. On the one side of the split, we have the sequential effect, which represents an ephemeral short-term effect, while on the other side of the split we have the mean RT–FP function and the FP-distribution effect, which represent a more endurable long-term effect.

The relative independence of long-term and short-term effects poses the challenge to account for these effects from a single integrative point of view. Both hazard-based preparation and trace conditioning fail in this respect, since the former cannot account for short-term effects, whereas the latter cannot adequately account for long-term effects (see **Table 2** for a summary of strengths and weaknesses). We now turn to possible solutions. First, we discuss the dual-process model, which takes the position that the short-term and long-term effects of FP are driven by different mechanisms. Then we present MTP, which conceives the short-term and long-term effects as different expressions of a single implicit learning mechanism.

**Table 2:** Summary of four explanations of key phenomena in temporal preparation (cf. **Figure 1**), and their strengths and weaknesses.

Explanation	Strengths	Weaknesses
***Hazard-based preparation***
Preparation is driven in accordance with the hazard function	Accounts well for long-term effects (e.g., FP distribution effect)	No explanation for short-term effects; unclear cognitive basis of hazard
***Trace conditioning***
Conditioned strength at critical moments is adjusted by inhibition during FP and activation upon S2 presentation	Accounts well for short-term effects (asymmetric sequential effect)	No adequate explanation for long-term effects; No explanation for relative independence of short-term and long-term effects
***Dual process model***
Combines hazard-based preparation with automatic carry-over of a refractory cost	Accounts for both short-term and long-term effects (**Figure 1**)	Disparate components for long-term and short-term effects; Unclear cognitive basis of the hazard-based component
***Multiple trace theory of temporal preparation (MTP)***
Combines dynamics of trace conditioning with storage in separate memory traces	Accounts for both short-term and long-term effects (**Figure 1**); Relies on well-established cognitive principles	Untested

## THE DUAL-PROCESS MODEL

Vallesi and Shallice (2007) proposed a dual-process model, which maintains the hazard function as an explanatory construct for the long-term FP phenomena and adds a short-term component to explain the sequential effect of FP. They envisaged the short-term component as an automatic “refractory” cost, which is proportional to the expenditure of preparatory resources on the preceding trial. The idea is that maintaining a high preparatory state over a relatively long FP depletes preparatory resources, which subsequently need time to recover. Therefore, when operating in isolation, the short-term component causes response on trial *n* to be slower as FP*_n_*_-1_ is longer, regardless of the duration of FP*_n_*. However, the short-term component typically does not operate in isolation, but co-occurs with the long-term component, representing controlled, hazard-driven preparation. It is assumed that hazard-driven preparation comes to dominate the preparatory state as FP*_n_* lengthens, thereby compensating the refractory cost. As a result, a strong sequential effect is observed at the shortest FP*_n_* where the refractory cost goes unopposed by hazard-driven preparation. As FP*_n_* increases, the growing influence of hazard-driven preparation gradually reduces the refractory cost to the point that nothing of it remains at the longest FP*_n_*. In short, according to the dual-process model, the sequential effect reflects a refractory cost inflicted by sustained preparation on trial *n* - 1, whereas its asymmetry is caused by an increasing dominance of hazard-driven preparation as FP*_n_* lengthens.

The combination of a short-term and long-term component endows the dual-process model with considerable explanatory power. The sequential effect is explained by the refractory component, the average RT–FP function across different FP distributions is explained by the hazard component, while the asymmetry of the sequential effect reflects the dynamic interplay of these components. That is, the model essentially explains all the key phenomena of **Figure 1**. Furthermore, by attributing the main effect of FP and the sequential effect of FP to the operation of different functional components, the model seems well equipped to explain the independent modification of these effects by a variety of experimental factors, as noted in the section on trace conditioning^4^.

In spite of these assets, this model can be seriously challenged on its theoretical assumptions. First, by maintaining the hazard function as an explanatory construct, the model inherits the key problem that it is unclear how participants acquire readily applicable knowledge about the changing hazard over time. Second, the assumed controlled nature of hazard-driven preparation is disputable because FP duration is a task irrelevant variable, which needs no monitoring for correct task performance. Attempts to empirically demonstrate a role for cognitive control during FP, by examining whether the RT–FP function becomes flatter when resources are supposedly withdrawn from the tracking of time during FP, have yielded mixed results (Van Lambalgen and Los, 2008; Steinborn and Langner, 2011; Vallesi et al., 2014). But even if cognitive control turns out to be an indispensible part of temporal preparation, it is still unclear whether it reflects hazard-driven preparation during FP or some other controlled process. For instance, it is conceivable that control must be exerted to keep the response system in check during FP, so as to prevent premature response – a process unrelated to the hazard function (Narayanan et al., 2006; Los, 2013). Third, the notion of resource depletion (the basis of the model’s short-term component) has a controversial status in experimental psychology, where it has been criticized as circular (e.g., Navon, 1984; Neumann, 1987) and impeding the discovery of more fundamental mechanisms (e.g., Pashler, 1994; Olivers and Meeter, 2008). Fourth, it remains somewhat opaque by what combination rule the two components interact. As long as no rule is specified, it is hard to assess what sequential effects the model actually predicts.

It seems possible to alleviate some of these problems without affecting the essence of the dual-process model. For instance, to solve the problem associated with resource depletion, one may conceive of the short-term component in terms of reinforcement at the imperative moment, followed by quick decay (e.g., Los, 2013; Yashar and Lamy, 2013). The short-lived reinforcement would then come to expression in the case of an intertrial repetition of short FPs, where it compensates for low hazard-driven preparation, but not in the case of an intertrial repetition of long FPs, where it is redundant to high hazard-driven preparation. Furthermore, there seems to be no compelling reason that hazard-driven preparation must be a controlled process, and this assumption can be dropped without altering the dual-process character of the model. However, the other two problems are more principled as they go to the essence of the dual-process model. That is, any variant of this model will have to specify how participants access the parameters necessary for estimating the development of hazard over time, as well as the way hazard-driven preparation interacts with the short-term component responsible for the sequential effect.

These critical notes aside, it should be noted that the dual-process model is the only model to date that accounts for all the phenomena shown in **Figure 1**. To achieve this feat, it sacrifices the unity of the family, by qualifying the sequential effect as a phenomenon that is fundamentally different from the long-term phenomena in temporal preparation (cf. **Table 2**).

## TOWARD A MULTIPLE TRACE THEORY OF TEMPORAL PREPARATION

The theory that we outline in this section belongs to a large class of multiple-trace theories, which have been very successful in explaining behavior in a great variety of tasks, including object categorization (Medin and Schaffer, 1978; Hintzman, 1986; Nosofsky and Palmeri, 1997), lexical decision (Logan, 1988, 1990), priming (Neill, 1997), visual search (Chun and Jiang, 1998; Hillstrom, 2000; Huang et al., 2004), and recently also time perception (Taatgen and Van Rijn, 2011). Multiple-trace theories share three basic assumptions. First, the attended elements constituting an episode are obligatorily stored in memory. It is typically assumed that an episode is demarcated by the outlines of a trial and that the stored elements include the intentions of the participant that applied during that trial (e.g., the task set), the relevant stimuli attended by the participant as well as the relevant actions. Second, each episode is stored as a single memory trace, which is added to an ever accumulating database of memory traces. Third, a new episode leads to a parallel retrieval of old memory traces to the extent that these traces contain elements (e.g., stimuli) that are currently attended. As a consequence, the repeated presentation of a stimulus in a given task context leads to an automatic retrieval of previous actions associated with that stimulus (e.g., Logan, 1988, 1990).

We develop MTP as an adjustment of the trace-conditioning model discussed earlier (cf. **Figure 2**). This is a natural way to proceed, because conditioning and multiple-trace theories share basic assumptions in the automatic retrieval of actions elicited by an attended stimulus. In particular, the idea that, after an acquisition phase, the presentation of a conditioned stimulus triggers a conditioned response, is perfectly consistent with the idea that an external event elicits an associated response by the obligatory retrieval of old memory traces. The main difference between these views is in the way experiences are stored in memory: Whereas in conditioning theory, it is usually assumed that the state of conditioning is committed to memory as a single, adjustable memory trace, in multiple-trace theory, each new experience is stored as a unique memory trace. The difference between these views can be easily bridged, however, and this is what MTP does.

## OUTLINES OF THE THEORY

Multiple trace theory of temporal preparation assumes that the dynamics on a single trial are the same as those described by the trace-conditioning model. That is, inhibition is applied during FP, to prevent premature response, while activation is released at the imperative moment when S2 is presented and responded to. However, instead of adjusting a state of conditioning, these processes are assumed to leave a more direct trace in memory, as they embody the preparatory experience. In particular, for each critical moment up till the imperative moment, the accumulated strength of inhibition and activation is stored in a single memory trace. It is furthermore assumed that the stored strength of activation has a lower maximum and a greater temporal dispersion as the imperative moment is more remote from S1, reflecting the growing time uncertainty as a function of FP (Klemmer, 1957). Other relevant events of a trial are also stored in the memory trace, including attended details of S1, S2, and the response with respect to S2. On each new trial, S1 serves as a retrieval cue for all relevant memory traces that were stored on earlier trials. At each critical moment during FP, these memory traces contribute to the preparatory state in accordance with their stored level of inhibition or activation for that moment. Finally, MTP assumes that newly formed memory traces are initially in a high state of activation, which decays over time toward a lower asymptotic level (cf. Taatgen and Van Rijn, 2011). As a result, the most recently formed memory traces contribute more strongly to current preparation than older traces. We refer to this activation level of the memory trace as *trace weight* to avoid possible confusion with the strength of activation at the imperative moment – an activation level that is stored inside the memory trace.

**FIGURE 3:**
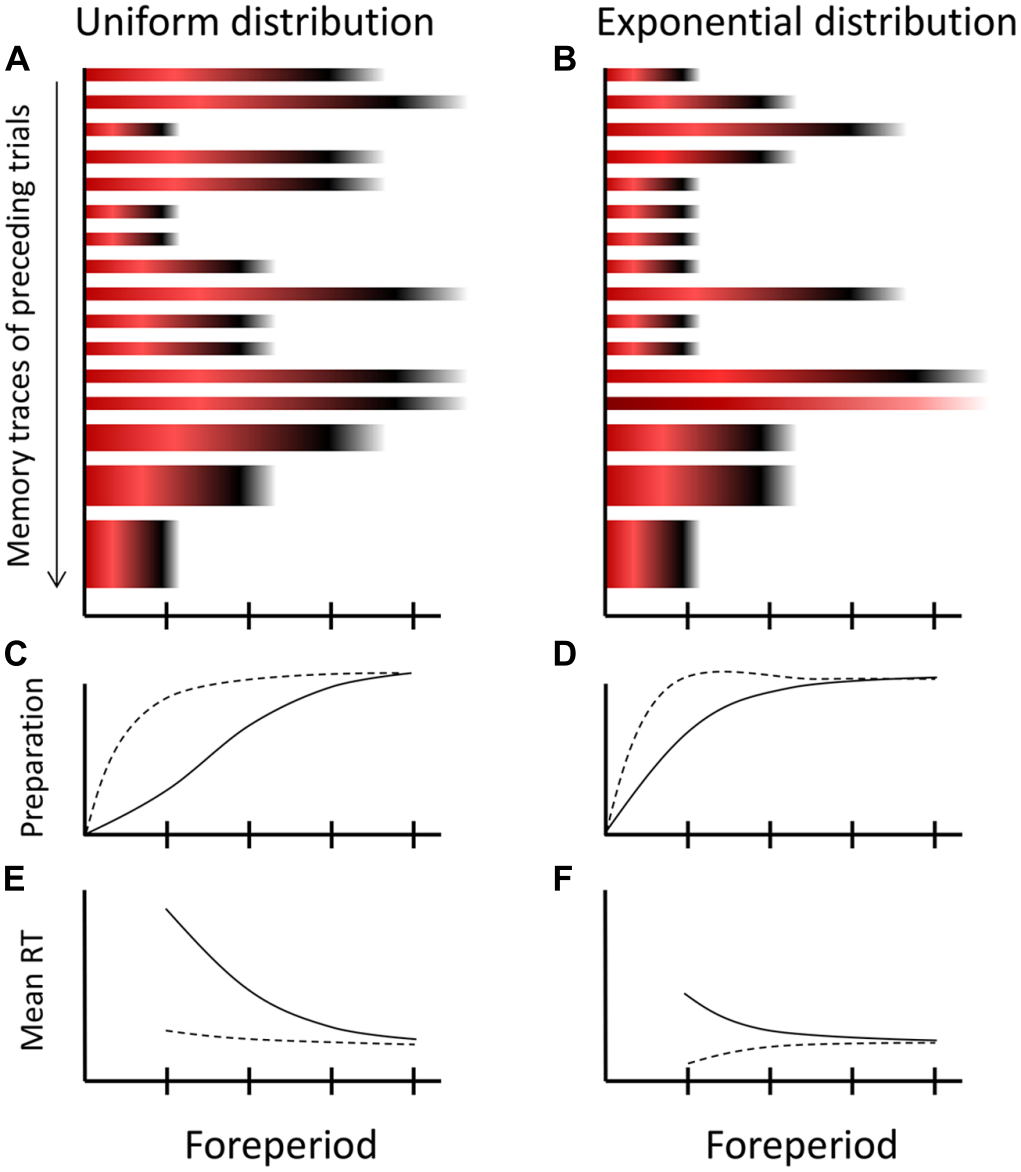
**Schematic overview of the multiple-trace theory of temporal preparation. (A,B)** Sixteen memory traces formed on subsequent trials under a uniform and exponential distribution of FPs. Each memory trace stores the preparatory experience of a trial in terms of accumulated inhibition during FP (shown by red) and activation at the imperative moment (shown by black), consistent with the dynamics of the trace-conditioning model (cf. **Figure 2**). Recently formed memory traces contribute more strongly to current preparation, as specified by their height. Note that under the exponential distribution, there is one catch trial, which lacks the activation component (black) in its trace. **(C,D)** The development of current preparation when all memory traces are weighted equally (solid line) and when weighted in accordance with their recency (broken lines). **(E,F)** Translation from preparation into observed mean reaction times (RTs). The curves in graphs **C–F** are all schematic.

The colored bars of **Figure 2** show the creation of a memory trace during a single trial according to MTP. Accumulated strength of inhibition is shown by red shades and strength of activation by black shades. Since the third critical moment was the imperative moment, relatively strong inhibition accumulated at the (passed) first critical moment (dark red), some inhibition at the second critical moment (light red), and activation at the imperative moment (black). The fourth critical moment is not stored in the memory trace, because the relevant episode was concluded by the response of the participant, that is, before the fourth critical moment was reached. **Figure 3** shows 16 memory traces that have been created over as many preceding trials under a uniform (**Figure 3A**) and exponential FP-distribution (**Figure 3B**). When comparing the different memory traces, note that activation is more dispersed over time as FP increases. Specifically, the dispersion of the black shades (activation) around the imperative moment on trials with the longest FP (e.g., **Figure 3A**, trial 2) is greater than on trials with the shortest FP (e.g., **Figure 3A**, trial 3). Also note that the relative weights of the traces are schematically depicted by their height. Specifically, the memory traces formed across the most recent trials (trials 14–16) are thicker than earlier formed memory traces.

Now, suppose that on the next trial (i.e., trial 17) the longest FP is initiated by the presentation of S1. At any moment during FP*_n_*, all previously formed memory traces contribute to preparation in proportion to (1) their stored inhibition or activation value for that moment and (2) their trace weight. For ease of exposition, we refer to the first contribution, when aggregated across trials, as activation-inhibition ratio (AI ratio). To obtain the ultimate level of preparation, differences in trace-weight should also be taken into account. Assuming that the older memory traces (formed more than, let’s say, three trials back) have approached an approximately equal asymptotic value, this simply means that the inhibition and activation values stored in the most recently formed memory traces contribute more strongly to current preparation than older traces.

The solid line of **Figure 3C** shows how temporal preparation would develop on the new trial 17 when it is exclusively driven by the AI ratio. As the first critical moment is approached during FP, the AI ratio is low (many more traces contain red than black at this point in time), yielding a low preparatory state. As time elapses further, the AI ratio gradually increases, because the contribution of aggregated inhibition gradually decreases relative to that of activation, thereby boosting preparation. By the time the last critical moment is reached, the contribution of inhibition has vanished, such that temporal preparation reaches its optimum. The broken line in **Figure 3C** shows how temporal preparation would develop if trace weights are taken into account. Here preparation reaches a high level even at the earliest critical moment, owing to the high trace weight of the most recently formed memory trace, which represents a trial on which S2 appeared at the earliest critical moment. Thus, despite a low AI ratio, preparation at a critical moment could still be high if a high activation level for that moment is stored in a memory trace with a high weight (i.e., a recently formed memory trace). Finally, **Figure 3E** shows how the level of temporal preparation translates into mean RT.

## ACCOUNTING FOR THE FAMILY OF FP EFFECTS

According to MTP, the family of phenomena in temporal preparation are all different expressions of the same mechanism. First, consider the effects of **Figure 1A**. The downward sloping mean RT–FP function in the variable-FP paradigm reflects the increasing AI ratio across subsequent critical moments as described above. The AI ratio is irrelevant in the constant-FP paradigm, because the same FP is repeated across trials, such that the preparatory state reaches its optimum at the imperative moment regardless the duration of FP. Here, the upward sloping RT–FP function reflects that the stored activation values are more dispersed over time as the imperative moment is more remote from S1. As a result, when activation values are aggregated across trials, the resulting preparation function has a greater spread and a lower maximum, consistent with the scalar property observed in animal timing (e.g., Roberts, 1981; Gallistel and Gibbon, 2000) as well as with notions of increasing time uncertainty as a function of FP (e.g., Klemmer, 1957; Niemi and Näätänen, 1981; Müller-Gethmann et al., 2003).

Second, the sequential effect of FP (**Figure 1C**) reflects that the weight of the most recently formed memory trace is relatively high, such that its stored preparatory experience contributes relatively strongly to current preparation. The asymmetry of this effect is explained in the same way as by the trace-conditioning model: Inhibition is stored for critical moments preceding but not following the imperative moment. Therefore, the level of preparation on trial *n* is reduced by inhibition on trial *n* - 1 if FP*_n_* is shorter than FP*_n_*_-1_ but not if it is longer. Finally, to the extent that a critical moment is passed by during FP, it becomes more strongly associated with inhibition and less strongly with activation that “spills over” from the imperative moment (see **Figure 2**, first versus second critical moment). This explains that RT on trial *n* is longer to the extent that FP*_n_*_-1_ was longer than FP*_n_*, an effect that is clearest for the shortest FP*_n_* (**Figure 1C**).

Third, the FP-distribution effect (**Figure 1B**) is mainly explained by differential AI ratios, as illustrated by **Figure 3** for the uniform and the exponential distribution. At the first critical moment, the AI ratio is much lower under the uniform distribution than under the exponential distribution, because memory traces with high inhibition values at this moment are far more frequent under the uniform distribution. However, this difference between distributions diminishes across subsequent critical moments (**Figure 3A** versus **Figure 3B**). Therefore, when abstracting from a possible compensating influence of the most recently formed memory traces, preparation is lower for the uniform distribution early during FP but catches up as FP increases (**Figure 3C** versus **Figure 3D**, solid traces), which explains the steeper average RT–FP function for the uniform distribution (**Figure 3E** versus **Figure 3F**). This aggregated effect across memory traces is strengthened by differential frequencies of specific intertrial transitions. In particular, a repetition of the shortest FP occurs far more often under the exponential distribution than under the uniform distribution. Thus, under the exponential distribution, preparation is more often boosted at the first critical moment through high trace weights, which further reduces the slope of the average RT–FP function.

Fourth, MTP has a straightforward solution for the relative independence of the short-term and long-term effects in temporal preparation. In particular, the short-term effect (the asymmetric sequential effect) is explained in terms of the *recency* of newly formed memory traces, whereas the long-term effects (average RT–FP function and the FP-distribution effect) are mainly explained in terms of the *relative frequency* of distinctive memory traces. Thus, any factor that selectively influences either the recency or the relative frequency of distinctive memory traces will selectively modify the short-term and long-term effects, respectively. For instance, the finding of Vallesi et al. (2013) that the duration of the intertrial interval attenuates the asymmetric sequential effect while leaving the average RT–FP function largely unaffected, would reflect that this variable influences the trace weights of recently formed memory traces while leaving the relative frequency of distinctive traces unaffected.

Finally, MTP also provides a natural account of effects of non-temporal features associated with FP (see Schröter et al., 2014; for a related idea and Thomaschke and Dreisbach, in press for an alternative account). For instance, in the time-event correlation paradigm, each specific S2-response pair has an equal probability of occurrence on each trial, but one pair is more probable after a short FP than after a long FP (e.g., 4/5 vs. 1/5), whereas these probabilities are reversed for another pair. It has been shown that the ensuing time-event correlation gets gradually expressed in behavior: Over the course of the experimental session, participants come to respond faster and more accurately to S2 when it occurs after its typical FP than when it occurs after its atypical FP (e.g., Miller and Schröter, 2002, Experiment 8; Wagener and Hoffmann, 2010; Thomaschke et al., 2011; Thomaschke and Dreisbach, 2013, in press). Meanwhile, participants remain generally unaware of this time-event correlation, as evidenced by self-report after the experimental session (e.g., Thomaschke et al., 2011; Thomaschke and Dreisbach, in press). These findings are readily accommodated by MTP, which assumes that the specific stimuli and responses of each trial get represented in the memory trace along with the duration of FP. As a result, memory traces with frequent combinations of FP and specific stimuli or responses will come to dominate traces with infrequent combinations of these features, thereby facilitating the corresponding behavior, consistent with the observations.

Our suggestion to account for time-event correlation effects in terms of MTP must be accompanied by a caveat. When S2-response pairs are defined by a compatible spatial relationship (as in **Figure 1**, top), intertrial repetitions of the specific S2 have been reported to yield slower responses than intertrial alternations of the specific S2 (e.g., Kirby, 1976; Soetens et al., 1985). MTP clearly predicts the opposite result, especially when FP is repeated in addition to the specific S2. Therefore, contributions other than those discussed here appear to be involved, such as a subjective expectancy of an alternation elicited by the preceding sequence of trials (e.g., Kirby, 1976; Soetens et al., 1985; Soetens, 1998; Jentzsch and Sommer, 2002) or inhibition of return (Posner and Cohen, 1984; Klein, 2000; Los, 2004). However, since repetition costs are the exception rather than the rule (e.g., Pashler and Baylis, 1991) they do not falsify the general principles of MTP.

In short, MTP provides a coherent explanation of the family of phenomena in temporal preparation shown in **Figure 1** as well as a framework for understanding patterns of dissociations between short-term and long-term FP effects. MTP also seems promising as an account of effects of non-temporal features associated with FP, such as the time-event correlation effect. Meanwhile, by conceiving all these effects as expressions of a single implicit learning mechanism, MTP reunites the family of phenomena in temporal preparation (cf. **Table 2**).

## COMPARISON TO OTHER MODELS

As noted earlier, MTP and the trace-conditioning model have similar assumptions about the within-trial dynamics of temporal preparation (i.e., inhibition during FP, activation at the imperative moment), but dissimilar assumptions about subsequent memory storage. The trace-conditioning model assumes that the within-trial dynamics influence a single adjustable memory trace, which makes it hard to account for long-term effects of FP (Los and Agter, 2005) and their selective modification by additional factors (e.g., Vallesi et al., 2013). This nearsightedness of the trace-conditioning model is solved by MTP, which assumes that old preparatory experiences are consolidated in independent memory traces.

The multiple-trace assumption of MTP is superior to the single-trace assumption of the trace-conditioning model also from a more general perspective of cognitive architecture. The learning rules of the trace-conditioning model imply that highly over-learned contingencies from the past can be completely overturned by a few recent trials with new contingencies. This makes any system based on old contingencies highly vulnerable to corruption, similar to removing a foundational brick from a building (Grossberg, 1987). MTP solves this problem because highly over-learned contingencies are consolidated in a multitude of memory traces, thereby safeguarding their stable contribution to future behavior. At the same time, MTP allows for flexibility when addressing novel contingencies by weighting new experiences more heavily than older ones. However, if the new contingencies turn out to be temporary, they will not have a strong bearing on future behavior as their weight will gradually diminish, such that the more numerous older memory traces will come to dominate again. In all, MTP seems to strike a good balance between stability and flexibility.

It is also interesting to compare MTP to the formalized pool model that Taatgen and Van Rijn (2011) proposed to account for phenomena of time perception. MTP and the pool model have similar assumptions about memory storage, but a different take on within-trial dynamics. The pool model adopts a timing device consisting of an accumulator that counts the number of pulses issued by a pulse generator since the onset of a time marker (Gibbon, 1977; see Buhusi and Meck, 2005; for a review, and Staddon, 2005, for a critique). After each trial, the number of pulses accumulated during FP is stored in a memory trace that starts contributing to the timing process on subsequent trials. Although it is conceivable that the pool model can account for the phenomena of temporal preparation just as well as MTP can, we postulated a different within-trial mechanism for two reasons. First, as noted earlier, there is considerable evidence for the within-trial dynamics postulated by MTP, in terms of inhibition during FP and activation at the imperative moment (see Los, 2013 for a review), so it makes sense to assume that these processes get represented in the memory trace. Second, it seems more in the spirit of the multiple-trace conception to assume that memory closely represents experience (e.g., preparation during FP) rather than its product (e.g., a number produced by a clocking device). Of course, these arguments are far from decisive, and future investigations may attempt to empirically distinguish between these views.

When compared to hazard-based explanations, MTP shares the point of view that long-term effects of temporal preparation cannot be explained as a mere propagation of short-term effects, as implied by the trace-conditioning model. However, beyond this general insight, the explanations differ fundamentally. Whereas hazard-based explanations need a separate mechanism to account for short-term effects, as elaborated in the dual-process model (Vallesi and Shallice, 2007), MTP attributes short-term and long-term effects to a single integrated learning mechanism at different stages of trace-decay. From a theoretical point of view, the position taken by MTP seems more parsimonious, because it needs not invoke a separate mechanism to account for different phenomena of the same family. Furthermore, whereas MTP is conceived in terms of well-known principles of learning and memory (e.g., Logan, 1988; Rosenbaum, 2014), the cognitive basis of hazard-based explanations remains to be specified, in particular with respect to how participants acquire knowledge of the hazard function.

It may seem possible to reconcile MTP and hazard-based explanations, by pointing at an apparent similarity between the hazard function and the changing AI ratio over time. Indeed, when considering the memory traces of **Figure 3**, it transpires that the AI ratio describes preparation in a way that mimics the hazard function. This is perhaps clearest for the exponential distribution (**Figure 3B**), where the AI ratio is approximately constant across critical moments (i.e., the proportion of red and black across memory traces is about equal at each critical moment), consistent with the constant hazard under this distribution. However, close examination reveals that this similarity is only approximate. The within-trial dynamics of MTP (**Figure 2**) imply that, as a critical moment is more remote from S1, it suffers less from inhibition when passed by during FP and it profits more from activation that spills over from a nearby imperative moment. As a result, by the AI ratio alone, the RT–FP function would still be downward sloping under the exponential distribution. It is only because of the preponderance of repetition trials with short FPs (where early critical moments profit from high activation values stored in traces with a high weight) that the average RT–FP function becomes flat according to MTP (see also **Figures 3D,F**). That is, according to MTP, the flat average RT–FP function is not a pure phenomenon, and emerges coincidentally from several contributing influences.

Moreover, the conceptual distance between AI ratio and hazard seems difficult to bridge. Within MTP, the AI ratio summarizes aggregated inhibition and activation across past memory traces. Crucially, it does not accomplish anything by itself; it is the information in the traces that drives preparation. That is, the AI ratio is a quantity that can be conveniently used to describe the state of preparation, but that does not play an active part in preparation at any stage. By contrast, on hazard-based explanations, the changing hazard during FP is supposed to be the driving force behind preparation. This means that participants must somehow derive the hazard function from experience on past trials, for instance, by using some mental tally system. By itself, this feat would still not have any impact on behavior as long as the derived hazard does not feed into a preparation process. Thus, whereas according to hazard-based explanations temporal preparation is newly created on each trial on the basis of changing hazard, according to MTP it reflects a dynamic re-enactment of preparation as stored in memory traces.

## A CRITICAL TEST AND FUTURE DIRECTIONS

To distinguish between MTP on the one hand and trace-conditioning and hazard-based accounts on the other hand, it would be imperative to dissociate overall trial history (the causal force in MTP) from both recent trial history (the driving force in trace conditioning) and current hazard (the driving force in hazard-based explanations). One could do that by examining transfer between blocks of trials with different FP distributions. According to both the trace-conditioning model and hazard based explanations, there should be hardly any transfer between blocks. Trace-conditioning does not predict any transfer, because of its nearsightedness. The state of conditioning is adjusted after each trial, so it should take only a few trials to wipe out any influence of a different preceding FP distribution. Hazard-based accounts make the same prediction, because participants should be capable to quickly tune in on the hazard function that applies in the new block, especially when advance information is provided on the FP distribution. By contrast, according to MTP, transfer between blocks with different FP distributions should be relatively persistent, because memory traces formed during one block cannot be undone when starting a new block.

If the outcomes of a transfer study support MTP, the obvious next step would be to formalize the theory and to fit it to the variety of data patterns that have been observed in temporal preparation studies since Woodrow’s (1914) seminal study one century ago. Apart from the family of phenomena that we considered in the present contribution, the theory should also account for the development of temporal preparation over very brief intervals (e.g., Müller-Gethmann et al., 2003; Los and Van der Burg, 2013) as well as for the selective modification of short and long-term effects of FP by additional factors. In the longer run, a more general theory of timing could be pursued, which provides an integrative perspective of phenomena in temporal preparation and time perception. The close kinship of MTP and the pool model (Taatgen and Van Rijn, 2011) inspires optimism about the feasibility of this objective.

## CONCLUSION

We have introduced a new theory of temporal preparation, MTP, which combines the within-trial dynamics of the trace-conditioning model (Los and Van den Heuvel, 2001) with memory storage in multiple traces (Taatgen and Van Rijn, 2011). MTP accounts for the family of FP phenomena (**Figure 1**) from a single integrative point of view, unlike alternative accounts based on the hazard function. Moreover, MTP has a solid cognitive basis in its reliance on well-established principles in learning and memory.

## Conflict of Interest Statement

The authors declare that the research was conducted in the absence of any commercial or financial relationships that could be construed as a potential conflict of interest.

## Footnotes

1 The inferred process has also been called alertness, temporal attention, temporal expectancy, non-specific preparation or simply preparation. The term temporal preparation seems to be most frequent in the recent reaction time literature.

2 The effects of FP shown in **Figure 1** are idealized to facilitate seeing the interrelationships among them. The many studies cited in the text attest to their robustness.

3 Given a constant hazard *p* across critical moments, the *a priori* probability that S2 will be presented at critical moment *k* (1, 2, 3, …) equals *p*(1 -*p*)*^k^*^-1^ (Nickerson and Burnham, 1969). Furthermore, to maintain a constant hazard for the last critical moment, an appropriate proportion of catch trials (i.e., trials without S2) should be added. Interestingly, response at the longest FP*_n_* has been found to slow down after a catch trial on trial *n* - 1 (e.g., Correa et al., 2004; Los, 2004; Los and Agter, 2005). This is consistent with the general pattern of sequential effects (**Figure 1C**), because a catch trial can be conceived as a trial with a long FP that extends beyond the latest critical moment.

4 Note, though, that the dual-process model does not always predict independence of the sequential effect of FP and the average RT–FP function, because at longer FPs, the short-term component is dominated by the hazard-driven component rather than being independent of it. In particular, factors that reduce the effectiveness of the hazard-driven contribution to the preparatory state should also reduce the asymmetry of the sequential effect of FP, because the refractory cost is then less strongly dominated at the longer FPs. We are aware of only one study in which this critical finding has been reported (Vallesi and Shallice, 2007).
